# Vitamin D and Gastric Cancer: A Ray of Sunshine?

**DOI:** 10.7759/cureus.18275

**Published:** 2021-09-25

**Authors:** Suchitra Shah, Zafar Iqbal, Mohammed G Alharbi, Harjeevan S Kalra, Megha Suri, Nitin Soni, Nkiruka Okpaleke, Shikha Yadav, Pousette Hamid

**Affiliations:** 1 Internal Medicine, California Institute of Behavioral Neurosciences & Psychology, Fairfield, USA; 2 Emergency Medicine, California Institute of Behavioral Neurosciences & Psychology, Fairfield, USA; 3 Internal Medicine/Emergency Medicine/Oncology, California Institute of Behavioral Neurosciences & Psychology, Fairfield, USA; 4 Pediatrics/Medicine, California Institute of Behavioral Neurosciences & Psychology, Fairfield, USA; 5 Psychiatry and Behavioral Sciences, California Institute of Behavioral Neurosciences & Psychology, Fairfield, USA; 6 Neurology, California Institute of Behavioral Neurosciences & Psychology, Fairfield, USA

**Keywords:** vitamin d, cholecalciferol, gastric cancer, ergocalciferol, vitamin d receptor, genetic polymorphism, vitamin d deficiency

## Abstract

Gastric cancer (GC) is one of the most aggressive malignancies, currently ranking third among cancers leading to death worldwide. Despite the recent advancements in GC research, it is most often diagnosed during the terminal stages and with limited treatment modalities contributing to its poor prognosis and a lower survival rate.

Much research has provided conflicting results between a vitamin D deficient status and the development of GC. Vitamin D is a well-known and essential hormone classically known to regulate calcium and phosphate absorption, enabling adequate mineralization of the skeletal system. However, the function of vitamin D is multidimensional. It possesses unique roles, including acting as antioxidants or immunomodulators while crossing the cell membrane, performing several intracellular functions, participating in gene regulation, and controlling the proliferation and invasion of cancer cells, including those of GC.

In light of this, it is imperative to analyze the causes of GC, review the factors that can be used to enhance the effectiveness of treatments, and discover the tools to determine prognosis, reduce mortality, and prevent GC development. In this review, we have summarized recent investigations on multiple associations between vitamin D and GC, emphasizing genetic associations, vitamin D receptors, and the prevalence of hormone deficiency in those developing this aggressive malignancy.

## Introduction and background

Gastric cancer (GC) is one of the five most common cancers to occur globally despite its abatement in incidence during recent years [1,2]. This aggressive malignancy manifests a poor prognosis due to its advanced stage of diagnosis and restricted treatment alternatives, placing GC third among cancers leading to mortality worldwide [3,4]. Furthermore, numerous risk factors such as genetics, *Helicobacter pylori* (most common), smoked foods, red meat, smoking, and alcohol also contribute to the development of GC [5-8]. Additionally, many investigations also support the significance of vitamin D in the overall pathogenesis of GC at both genetic and molecular levels.

**Figure 1:**
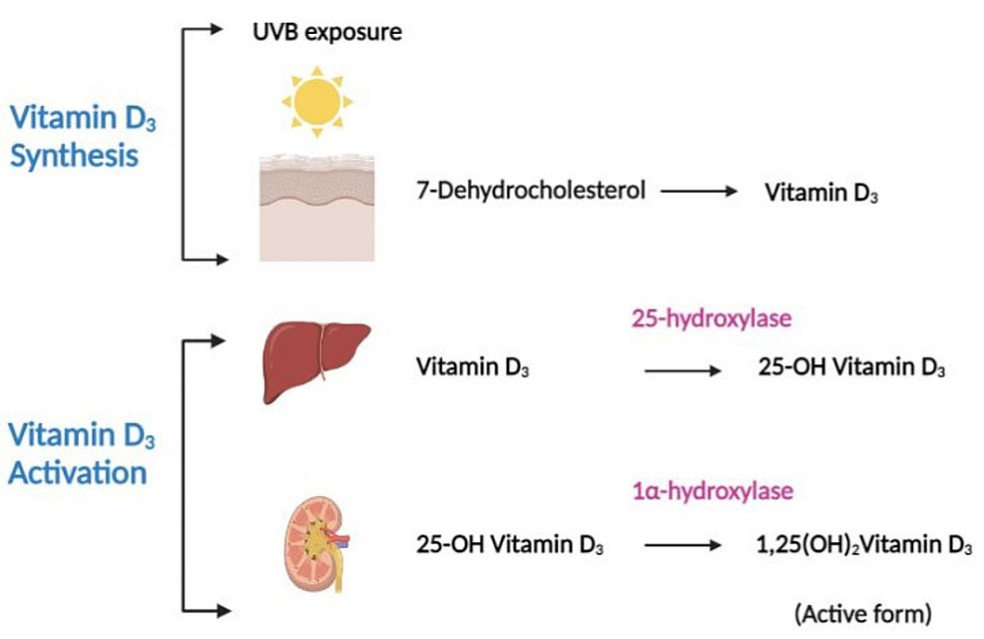
Schematic representation of the dermal synthesis of vitamin D3 and its activation. Under the effect of ultraviolet B light from the sun, vitamin D_3_ is produced in the dermis from 7-dehydrocholesterol. Following production, it enters the liver, where the enzyme 25-hydroxylase converts it to 25-hydroxyvitamin D_3_ (25-OH Vitamin D_3_). It subsequently travels to the kidneys, where it is transformed into 1,25-dihydroxyvitamin D_3_ (1,25(OH)2Vitamin D_3_), the physiologically active form of vitamin D. [Original illustration]

Despite its essential properties as a fat-soluble vitamin, vitamin D is also an active steroid hormone. Its primary function is bone mineralization by regulating calcium and phosphorus homeostasis. We can obtain vitamin D from various foods, supplements and via dermal production under the influence of sun exposure. It is available in two forms, vitamin D_2_ (ergocalciferol) and vitamin D_3_ (cholecalciferol). However, since both forms are inactive, they must be hydroxylated twice before activating the final product. The initial activation happens in the liver, followed by the second in the kidneys by enzymes 25-hydroxylase and 1alpha-hydroxylase (1α-hydroxylase) respectively to produce 1,25-dihydroxyvitamin D_3_ (1,25(OH)2Vitamin D_3_) (active form), which exerts the physiological effect [6,9,10]. Figure 1 shows the formation and activation of vitamin D_3_.

Moreover, many studies have highlighted the active participation of vitamin D in other processes like the immune system, inflammation, gene regulation, signal transduction, and finally, the development of cancer. Epidemiological and animal studies mentioned earlier showed that vitamin D exerts an antineoplastic effect via its vitamin D receptor (VDR). Its interaction stimulates apoptosis and differentiation while inhibiting invasion, angiogenesis, proliferation, inflammation, and metastasis [11,12]. In addition, the VDR functions as a transcription factor, regulating several gene expressions.

Since vitamin D is essential in the control and maintainance of many of the crucial tasks within the human body, its deficiency can contribute to immune dysregulation. In infections like *Helicobacter pylori*, vitamin D deficiency can lead to the failure of its removal [13,14]. Similarly, GC patients may demonstrate a greater survival rate with adequate vitamin D levels as compared to individuals with VDD [10], suggesting that vitamin D could be a powerful constituent in the GC mechanism. This review focuses on various associations between vitamin D and the pathogenesis of GC, which may be beneficial for early diagnosis and treatment. Overall, it provides an understanding of delaying the progression of GC and lowering its associated mortality rate.

## Review

Methods

The literature was searched in the PubMed database. The regular keywords used in the search for vitamin D are as follows: Vitamin D, Cholecalciferol, Calcitriol, Drisdol, 1,25dihydroxycholecalciferol, Ergocalciferol, VitaminD2, VitaminD3; For GC, we used keywords such as Stomach cancer, Stomach neoplasm, Stomach tumor, Stomach carcinoma, Gastric carcinoma, Gastric cancer, Gastric tumor, Gastric neoplasm. The Boolean search strategy was applied using "OR" in the regular keywords, giving 92,292 and 161,726 results for vitamin D and GC, respectively. Regular keywords were then combined using the Boolean term "AND" that generated 1,48,274 papers. We also used Medical Subject Headings (MeSH) keywords such as “Vitamin D” and “Stomach Neoplasms” that produced 61,722 and 99,898 articles, respectively. The Boolean term “AND” was implemented on MeSH keywords, which gave us 48 papers. Finally, both regular and MeSH keywords together yielded 119 articles.

Results

Only studies in the English language were included, which reduced the number of articles from 119 to 113. These 113 papers were screened based on the relevant topic, and any articles with animal studies were excluded. Fifty articles were retrieved that were either free full text or abstracts. Table 1 and Table 2 present a summary of the most relevant study characteristics.

**Table 1:** A table outlining the studies exploring the genetic involvement of vitamin D in gastric cancer. BMP3: Bone morphogenetic protein 3; GC: Gastric cancer; Hh: hedgehog; miR: miRNA; PTEN: Phosphatase and tensin homolog deleted on chromosome 10; VD: vitamin D; VDR: vitamin D receptor; VDD: vitamin D deficiency; VDBP: vitamin D binding protein

Author	Country	Year	Study design	Key findings
Pan et al. [15]	China	2010	Experimental	VD, in combination with trichostatin A/sodium butyrate and 5-aza-2’deoxycytidine, promotes apoptosis in gastric cancer cells via raising PTEN expression.
Baek et al. [16]	Korea	2011	Experimental	VD reduces gastric cancer cell survival by suppressing Hh signaling and is synergistic with anti-cancer drugs (Adriamycin, Vinblastine, and Paclitaxel).
Cong et al. [17]	China	2015	Case-Control	VDR FokI polymorphism and GC risk have a positive relationship.
Chang et al. [18]	China	2015	Experimental	VD restricts gastric cancer cell proliferation by stimulating miR-145 expression.
Zhao et al. [19]	China	2019	Experimental	VD increases BMP3 expression and delays GC progression.
Parsamanesh et al. [20]	Iran	2019	Case-Control	A negative association has been observed between VDR FokI polymorphism and GC risk, whereas VDR TaqI polymorphism has shown a positive relationship with GC.
Durak et al. [21]	Turkey	2019	Case-Control	GC risk has not been correlated with VDBP, TaqI, and FokI VDR polymorphisms; VDD deficiency increases GC risk.
Chang et al. [22]	China	2019	Experimental	VD inhibits GC cell proliferation by upregulating miR-99b-3p expression.
Calcagno et al. [2]	Brazil	2019	Review	VD produces an anti-cancer effect in gastric cancer by regulating histone acetylation.
Qadir et al. [23]	India	2021	Case-Control	The BsmI VDR polymorphism has been linked to GC risk; ApaI and TaqI VDR polymorphisms have not been attributed to GC risk.
Hoseinkhani et al. [24]	Iran	2021	Case-Control	FokI VDR polymorphism is associated with GC risk; However, TaqI, ApaI, and BsmI VDR polymorphisms are not related to GC risk.

**Table 2:** A table summarizing the association of vitamin D deficiency with gastric cancer. GC: gastric cancer; VD: vitamin D; VDD: vitamin D deficiency; VDR: vitamin D receptor.

Author	Country	Year	Study design	Key findings
Ren et al. [10]	China	2012	Retrospective-Observational	VDD has been linked to a poor prognosis of gastric cancer.
Bao et al. [7]	China	2014	Experimental	VD and Cisplatin promote apoptosis and cell cycle arrest in gastric cancer cells in a synergistic fashion.
Wen et al. [25]	China	2015	Experimental	VDR expression is lowest in gastric cancer compared to precancerous and normal gastric tissue.
Khayatzadeh et al. [26]	Iran	2015	Meta-analysis	GC has no relationship with VD level or consumption.
Vyas et al. [8]	USA	2016	Case-Control	VDD is correlated with a higher risk of gastric adenocarcinoma.
Du et al. [27]	China	2017	Review	The risk and mortality from gastric cancer are higher in VDD.
Yildirim et al. [14]	Turkey	2017	Prospective-Observational	VDD is correlated with the failure of Helicobacter pylori elimination.
Parizadeh et al. [28]	Iran	2019	Review	Ultraviolet B radiation reduces the risk of gastric cancer; VDD increases the risk of mortality in GC.
Kwak et al. [29]	Korea	2020	Cross-Sectional	VDD is considered a risk factor for GC.
Hedayatizadeh-Omran et al. [30]	Iran	2020	Case-Control	An increased prevalence of VDD has been found in GC; VDD is more prominent in high-grade GC.

Genetic role of vitamin D in gastric cancer

Genetic Polymorphism 

Vitamin D is a vital hormone synthesized for the adequate functioning of normal and healthy tissues. It performs its function by binding to VDR plus one of the retinoid X receptors (RXR) to produce a complex. The complex translocates inside the cell nucleus and binds to vitamin D response elements (VDRE) to further regulate the transcription of its target genes [31]. The gene encoding VDR is located on chromosome 12q13.1 [32,33]. Several *VDR* gene polymorphisms are linked to cancers, including colon, breast, ovarian, prostate cancer, and melanoma [34]. The most common* VDR* gene polymorphisms along with their location are: FokI (rs2228570) in exon2 in the 5’ end of the *VDR* gene [35], and TaqI (rs731236) in exon9, BsmI (rs544410), and ApaI (rs7975232) in intron 8 of the 3’ end region of the *VDR* gene [36,37]. Numerous studies have mentioned the association of these polymorphisms with the development and progression of GC.

Cong et al. demonstrated the relationship between the FokI polymorphism of the *VDR* gene to the risk of developing GC. In the FokI polymorphism, the nucleotide ATG becomes substituted with ACG in the first start codon, where translation begins. The latter results in the allele change from “f” to “F.” The f allele, when compared to F, bears an association with an increased risk of GC, a higher level of c-reactive protein (CRP), and more inadequate GC differentiation, contributing to the poor prognosis produced by GC [17]. Similarly, another study describes a positive correlation between the FokI polymorphism with GC susceptibility (p= 0.021). In contrast, other *VDR* polymorphisms (BsmI, ApaI, and TaqI) have no significant associations with the risk of GC compared to the healthy groups [24].

However, Parsamanesh et al. found that rather than FokI, the TC genotype of TaqI *VDR* polymorphism is related to the risk of GC (p = 0.002, OR: 2.39) [20]. Likewise, Durak et al. observed that FokI and TaqI polymorphisms of the *VDR* did not correlate with the susceptibility of GC (p > 0.05), yet found a higher number of the t allele of the TaqI variant in the advanced stage GC, suggesting a nexus between the t allele and a poorer prognosis for GC. In their research, authors assert that VDD strongly correlates with increased GC susceptibility, but the gene polymorphism of vitamin D binding protein (VDBP; rs7041) does not [21]. In addition, another investigation discovered that the BsmI polymorphism is strongly related to GC development, particularly in patients with a high BMI, yet ApaI and TaqI are not. Additionally, ApaI, TaqI, and BsmI variants of the *VDR* gene markedly limit GC survival [23].

Epigenetics

Calcagno et al. describe the effect of vitamin D on epigenetics. Epigenetics refers to a heritable trait that induces alteration in gene expression without changing the DNA sequence [38]. One of the unique epigenetic mechanisms is histone post-translational alterations. Histone acetylation and DNA methylation have a significant impact on cancer prognosis and treatment results. Histone acetylation results from a dynamic equilibrium between histone acetyltransferases (HATs) and deacetylases (HDACs). Histone acetylation causes gene activation, whereas its deacetylation leads to the silencing of genes. HDAC expression is high in GC, causing a lower expression of tumor suppressor genes (TSGs). Interestingly, vitamin D activates HATs and boosts the expression of TSGs, serving as an anti-cancer agent by modifying epigenetic pathways, which VDRs usually regulate in GC [2].

Pan et al. established the relation between vitamin D and the TSG phosphatase and tensin homolog deleted on chromosome 10 (*PTEN*). Vitamin D enhances GC cell death by triggering *PTEN* upregulation through VDR-mediated suppression of the *PTEN* promoter methylation. VDR synergistically stimulated PTEN overexpression with transcription factors like early growth response gene-1 (*Egr-1*) and HAT (P300). Additionally, vitamin D amplifies *PTEN* expression and accelerates apoptosis in GC cells, particularly when accompanied by epigenetic modifiers like HDAC inhibitors (trichostatin A/TSA and sodium butyrate) and DNA methylation inhibitors (5-aza-2’deoxycytidine/5aza). These data suggest that vitamin D coupled with epigenetic modifiers could benefit patients with GC as a promising treatment modality [15].

According to Zhao et al., cancer pathogenesis involves the blockage of several TSGs due to their promoter's methylation. Bone morphogenetic protein 3 (*BMP3*) is a known TSG, downregulated and expressed in low quantities in GC, owing to promoter hypermethylation. Moreover, VDRE is present in the methylation region of the *BMP3* gene promoter. Vitamin D, along with its receptor, is found to bind to these VDREs (p= <0.05), inhibiting *BMP3* promoter methylation and increasing the expression of the TSGs in gastric cancer cells. The apparent strength in association suggests the relation between vitamin D and the *BMP3* gene, plus the anti-cancer effect of vitamin D in GC via suppression of *BMP3* methylation [19].

Vitamin D regulation and cancer progression

Vitamin D has been related to cancer through many signaling mechanisms [39]. The hedgehog (Hh) signaling system has been associated with the advancement of many cancers, including GC [40]. An experimental investigation uncovered a relationship between vitamin D and the Hh signaling system, promoting the survival of GC cells. GC cells treated with vitamin D lowered Hh signaling target genes, signifying that the vitamin can weaken the GC cell survival. Furthermore, vitamin D acts synergistically with anti-cancer drugs such as Adriamycin®, paclitaxel, and vinblastine, thereby increasing the survival of GC patients [16].

Vitamin D regulates several genes, including micro RNAs (miRNA), which play a significant role in cancer development [41-43] and anti-tumor activities [44,45]. miRNAs are chief regulators of messenger RNA (mRNA) and can potentially modify the cell cycle, cell proliferation, cell invasion, and apoptosis. Alteration of specific miRNA can lead to cancer development, partially due to their behavior as oncogenes or TSGs in cancer cells. GC shows lower miRNA-145 (miR-145) than normal gastric tissue [46]. Chang et al. describe vitamin D's effect on miR-145 expression in GC cells. The authors observed that vitamin D promoted miR-145 expression, resulting in expanding cells in the S-phase and decreasing cells in the G2/M-phase of the cell cycle, explaining that miR-145 prevented the S to G2 transition in GC cells in vitro. Vitamin D also inhibits E2F transcription factor 3* (*E2F3) found to be upregulated in GC, cyclin-dependent kinase 6 (CDK6), prime targets of miR-145, and the subsequent E2F3 regulated cell cycle genes such as cyclin-dependent kinase2 (CDK2) and cyclinA2 (CCNA2). As a result, vitamin D can exert its anti-cancer activity via overexpression of miR-145 [18].

**Figure 2:**
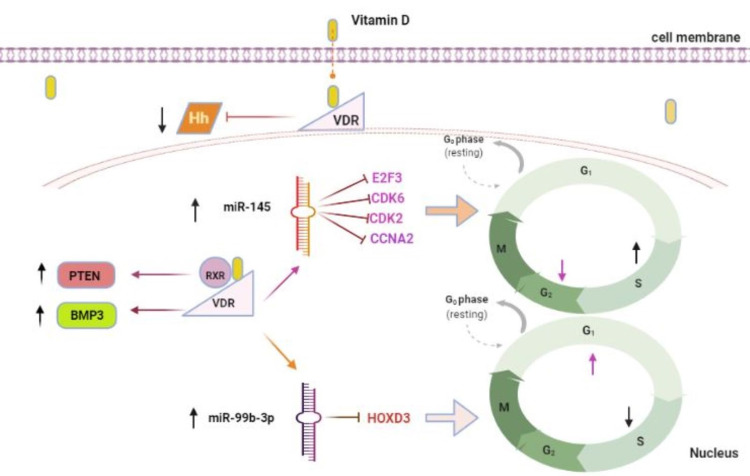
The anti-cancer effect of vitamin D is mediated by the vitamin D receptor. Vitamin D upregulates the tumor suppressor genes (*PTEN* and *BMP3*), causing GC cell death. It blocks the Hh signaling system, reducing GC cell viability. Vitamin D increases the expression of miR-145 and inhibits E2F3, CDK6, and the subsequent cell cycle regulatory genes CDK2 and CCNA2, causing the arrest of the S to G2 transition of the GC cell cycle. Additionally, vitamin D inhibits HOXD3 and upregulates the miR-99b-3p expression resulting in the cessation of G1 to S transition of the GC cell cycle. VDR: vitamin D receptor; GC: gastric cancer; RXR: retinoid X receptor; *PTEN*: phosphatase and tensin homolog deleted on chromosome 10; *BMP3*: bone morphogenetic protein 3; Hh: hedgehog; E2F3: E2F transcription factor3; CDK: cyclin-dependent kinase; CCNA2: cyclinA2; HOXD3: homeobox D3; miR: miRNA. [Original illustration]

Chang et al. have additionally revealed another vitamin D-regulated miRNA (miR-99b-3p). They discovered that like miR-145, miR-99b-3p was reduced in GC cells and that VDR increased miR-99b-3p expression in GC cells. Unlike miR-145, vitamin D and miR-99b-3p inhibit homeobox D3 (HOXD3) protein, primarily expressed in GC cells. In contrast to miR-145, miR-99b-3p increases cells in the G1 phase and reduces them in the S phase, implying that miRNA-99b-3p prevents the G1-S transition in the GC cell cycle [22]. VDR-mediated miRNA regulation appears to be one of the critical mechanisms in vitamin D's antiproliferative activity based on the studies mentioned above. Figure 2 illustrates the anti-tumor action of vitamin D.

Vitamin D deficiency and GC association

Several solid tumors (particularly the stomach, colon, liver and gallbladder, pancreatic, lung, female breast, prostate, bladder, and kidney cancers) appear to be reduced by adequate vitamin D levels [47]. Ren et al. measured vitamin D in GC and discovered that 8.1% of patients had sufficient levels (>75nmol/L), 34% had inadequate levels (50-75 nmol/L), and 57.9% had deficient levels (50nmol/L). Vitamin D level was also inversely related to tumor staging and lymph node metastasis, while tumor size, position, differentiation, and distant metastasis had no significant relationship. Furthermore, the five-year survival rate was 57.8% in the group with high vitamin D levels and 43% in the group with low vitamin D levels; vitamin D levels were indicated to be an independent prognostic factor and were comparable to bad prognosis in GC [10].

As VDR mediates vitamin D activity, Wen et al. evaluated the expression of VDR in normal, precancerous, and cancerous gastric tissues. They compared it with the clinicopathological characteristics of GC patients; They found VDR expression was markedly reduced in GC tissues versus healthy and precancerous tissues; secondly, well and moderately differentiated tissues demonstrated profound VDR expression in contrast with the poorly differentiated ones. Finally, VDR was expressed in large quantities in smaller gastric tumors than larger ones [25].

Vyas et al. concluded that the prevalence of VDD in patients with gastric adenocarcinoma was significantly higher when compared to patients with normal vitamin D levels (83.7 % vs. 63.27 %), suggesting a significant relationship between VDD and gastric adenocarcinoma. However, they declared no association between the degree of deficiency and the staging of GC like the above study [8].

To further understand the impact of vitamin D in GC, various studies with distinctive designs have been performed. The majority of ecological investigations using ultraviolet B exposure (UVB) reduced the incidence and mortality of GC [27,28]. A recent study by Kwak et al. discovered that patients with low vitamin D levels have a higher predilection for developing GC. They observed that the odds ratio (OR) for GC was 0.52 (95% CI: 0.30, 0.92) in the arm with increased total vitamin D levels (≤ 20ng/mL), compared to the decreased total vitamin D levels (<12ng/mL) with a p-value of 0.030; This signifies that higher vitamin D levels have a lower prevalence of GC and vice versa [29].

Hedayatizadeh et al. noticed a higher prevalence of VDD in GC cases than controls, with an evident decline in vitamin D concentration in high-grade GC cases. However, no correlations were found between lymph node metastasis, distant metastasis, tumor site, and vitamin D concentration [30]. On the contrary, in a meta-analysis conducted by Khayatzadeh et al., no significant relationship between vitamin D intake/serum vitamin D status and GC development was elucidated [26]. Consistent with this, a few more further studies revealed no correlation between vitamin D level and risk of GC [9,48].

The function of vitamin D in the immune system is widely understood. Numerous investigations have established the relation between VDD and infectious diseases [49, 50]. Yildirim et al. disclosed the relationship between the failure of eliminating HP in vitamin D deficient patients. In their study of 220 patients, elimination was achieved in 170 patients (77.2%), and 50 patients (22.7%) had a failure, with mean vitamin D levels substantially lower in the elimination failure group than in the successful treatment group (9.13 ± 4.7 vs. 19.03 ± 8.13; p= 0.001) [14]. A recent study that supports this notion was published by Yang et al., including results that patients with VDD had a slower elimination rate of HP (OR=0.09; 95%CI=0.2,0.4). Moreover, they reported that the average vitamin D level was higher in HP-negative individuals than the HP-positive individuals [13]. Based on the above findings, VDD could be a potential cause for the failure to eliminate HP, and adequate levels of vitamin D might be beneficial for the effective elimination of the infection.

On the other hand, Bao et al. reported the synergistic action of vitamin D and anti-cancer drug, cisplatin, against GC. Vitamin D amplified the effect of cisplatin by upregulating the pro-apoptotic protein like BCL2-associated X protein (Bax), enhancing cell cycle regulators such as p21 and p27 [51], and reducing the phosphorylation of phosphatidylinositol 3-kinase/AKT and extracellular-signal-related kinase 1/ERK, kinases implicated in GC cell proliferation and apoptosis [52,53]. Vitamin D potentiates the anti-cancer action of cisplatin by controlling cell proliferation, promoting apoptosis, and arresting GC cells in the G0/G1 phase of the cell cycle [7].

Limitation

Certain limitations exist in our study as the data was gathered only from one database (PubMed); only studies published in English were selected; studies with free full text and pertinent abstracts were solely included, and most of the collected studies were only in vitro studies.

## Conclusions

Several studies have been conducted to explore the relationship between vitamin D and GC. We observed a variety of correlations between vitamin D and GC. In most studies, GC patients have shown an increased prevalence of VDD, although few studies on VDD prevalence in GC patients are paradoxical. UVB radiation has been shown in most ecological studies to lessen the incidence and mortality of GC. An adequate vitamin D level has been associated with an increase in the survival rate of GC patients, and a low vitamin D level can be considered as a poor prognostic factor in GC.

Furthermore, variations in the *VDR* gene have been attributed to an increased risk of various malignancies, including GC. Many *VDR* gene polymorphisms are associated with the risk of GC. However, various research on *VDR* gene polymorphisms and GC risk have yielded inconsistent results.

Vitamin D exerts an anti-cancer effect by different mechanisms, such as regulating epigenetic pathways, upregulating the expression of miRNAs, boosting the action of cisplatin, stimulating TSGs, and regulating intracellular signal transduction. We also found that serum vitamin D is lower in the HP-positive patients than negative ones, and vitamin D deficient patients fail to eliminate HP. Based on the facts presented above, we may conclude that vitamin D is a protective factor in GC. It can be utilized as a promising technique to treat GC and increase survival rates by correcting the deficiency of vitamin D. However, additional studies are required to fully assess the genetic association of VDR in GC for a more profound knowledge of how to diagnose and treat the aggressive malignancy early and effectively to maximize the survival outcomes.
